# Experiences of Racism Associated with Self-Reported Hypertension Among African American/Black Individuals

**DOI:** 10.1007/s40615-025-02632-1

**Published:** 2025-09-16

**Authors:** Jacquelyn V. Coats, Kia L. Davis, Erin L. Linnenbringer, Taylor Bass, Vetta L. Sanders Thompson

**Affiliations:** 1 Center for the Study of Aging and Human Development, Duke University School of Medicine, Durham, NC, USA; 2 School of Medicine, Department of Surgery, Division of Public Health Sciences, Washington University in St. Louis, St. Louis, MO, USA; 3 Saint Louis Behavioral Medicine Institute, St. Louis, MO, USA; 4 Brown School, Washington University in St. Louis, St. Louis, MO, USA

**Keywords:** Black, African American, Racism, Hypertension, Discrimination, Health disparities

## Abstract

**Purpose:**

This paper sought to understand how experiences of various levels of racism across multiple domains and geographical regions were associated with higher odds of hypertension among African American adults by sex.

**Methods:**

Data were from the African American Identity, Socialization, Discrimination, and Outcomes Project, collected January–February 2020. Participants self-identified as African American/Black individuals, were 18 + years of age, and completed a Qualtrics survey with items measuring perceived experiences of racism and health. Hypertension diagnosis served as the outcome variable. The Experience of Discrimination Questionnaire assessed racist experiences in six areas: personal (insults, slurs), education, employment, housing, law, and police interaction. We conducted bivariate analyses examining the distribution of experiences of discrimination by age, sex, and region using logistic regression to understand how racism patterned by these factors impacted odds of hypertension.

**Results:**

In our sample (*n* = 1177), the distribution of racism experiences varied by sex and age. Additionally, reporting job discrimination and having a usual source of care were significantly associated with higher odds of hypertension in the overall sample (OR_Job_ = 1.44, 95% CI: 1.04–1.99; OR_care_ = 1.73, 95% CI: 1.26–2.40). Females with a college or graduate degree reported slightly lower odds of hypertension (OR_Edu_ = 0.52, 95% CI: 0.32–0.89).

**Discussion:**

People reported experiencing racism in different domains as they aged. Findings indicate sex-based differences in the experiences of racism and its association with hypertension. Our study underscores the importance of understanding which experiences of racism are more salient, for whom, when, and with what impact.

## Introduction

Racism is enacted through attitudes, beliefs, behaviors, and policies that lead to inequity in the treatment of individuals and communities as well as the structuring of resources, power, and opportunity [1]. Racism exists and operates at many levels, but much of the research into its health impacts does not always acknowledge or address these levels [2, 3]. A growing emphasis on health equity suggests the need to improve related research, as evidenced by the 2020 Presidential Advisory Call from the American Heart Association that called for accelerated research on the “joint effects of multiple domains of racism, including structural, interpersonal, cultural, and anti-Black” [4].

*Structural racism* is defined by Gee and Ford as “the macro-level systems, social forces, institutions, ideologies, and processes that *interact* with one another to generate and reinforce inequities among racial and ethnic groups” [5]. Structural racism has been enacted against Black people in the USA since the transcontinental slave trade through the Black Codes, Jim Crow laws (late nineteenth and early twentieth century laws enforcing segregation), and modern discriminatory actions such as mass incarceration, continued housing discrimination, and more [6]. Structural racism restricts African American individuals’ freedom [7, 8] and access to resources [6, 9].

*Institutional racism* refers to the discriminatory policies, practices, and programs within institutions; manifests as differential access to resources and opportunities; and involves materials (housing, education, employment) and power (knowledge, wealth, access to organizational infrastructure) [3, 10, 11]. These institutional practices and policies reinforce each other and intertwine to contribute to current residential segregation and overexposure to health-damaging social and environmental risk factors in Black communities [12]. The disproportionate burden of underfunded education, lack of healthcare access, and the lack of high-quality foods in Black communities are manifestations of institutional racism.

*Interpersonal racism* is the most familiar and one of the more studied forms of racism [11]. It references intentional and unintentional actions and can include avoiding members of certain groups, disrespectful interactions, bias toward individuals based on group membership, and others. While this form of racism may not produce population effects, it is not without individual impact.

Scholars have identified pathways by which interpersonal racism may harm health, such as adverse physical, social, and economic exposures, and maladaptive coping behaviors [13]. Studies, including systematic reviews, indicate that the strongest, most consistent findings are that interpersonal racism is associated with negative mental health outcomes and health behaviors (dietary patterns, sleep patterns, and substance use) [13]; weak associations exist with self-assessed health status and physical health outcomes [5, 14, 15]. Studies have demonstrated associations between perceived interpersonal and institutional racism and hypertension and nighttime ambulatory blood pressure among African American adults and adolescents [16–19]. Exposure to institutional racism also has been linked to lower subjective cognitive function [20].

A growing body of research attempts to assess exposure to structural racism and its health impact [21–24]. Research has linked state-level structural racism to increased odds of myocardial infarction for Black people but null or inverse effects for White people [25]. Structural racism also has been linked to premature mortality for Black adults in Jim Crow states [26] and to higher odds of having aggressive subtypes of breast cancer for Black women in Jim Crow states [27]. Krieger and colleagues found that Black participants born in a Jim Crow state had a higher odds ratio for hypertension than their Black peers born elsewhere, but the relationship was attenuated with the addition of covariates such as body mass index and education [22].

Considering the synergistic ways that different forms of racism interact to impact health, we used survey data (collected before COVID-19) to deepen our understanding of how experiences of racism influence health status. Specifically, we examined experiences of racism at the interpersonal, institutional, and structural level and their effects on hypertension.

## Methods

### Study Procedure

All participants in the African American Identity, Socialization, Discrimination, and Outcomes Project were recruited between January 2020 and February 2020 through Qualtrics Panel services. Participants were invited to participate in a Qualtrics-administered online survey exploring racial identity and factors associated with it. We followed Qualtrics best practices recommendations. The total sample size was 1623 individuals. The median survey completion time was 13.3 min, but it could require 35 min to complete. Individuals who completed the survey in half or less the median time were screened out of the sample as excessively rapid completion is considered a sign that respondents chose answers at random to get through the survey quickly; as well as those who did not complete at least 80% of the survey (*n* = 347). Incentives for participation were unique to each individual and based on their Qualtrics participation agreement. This study was approved by the Washington University in St. Louis Institutional Review Board.

### Eligibility and Recruitment

A total of 1177 men and women, 18 years of age or older, who self-identified as African American/Black participated in the study. Inclusion criteria were native born and currently residing in the USA. Exclusion criteria were being aged 17 years old or younger, having a racial/ethnic identity other than African American/Black, and being unable to read English. The panel survey enforced sample quotas: region—55%, South; 17%, Northeast; 18%, Midwest; and 10%, West. The panel enforced sample quotas were intended to conform to the African American/Black population percentages in each region at the time of data collection. The remaining sample composition was sex 51% female and 49% male; age—35% less than 35 years, 50% 35–64 years old, and 15% 65 years or older; education—40.8%, high school diploma, GED, or less; 20.9%, some college (no degree); and 38.3% associate’s degree or higher; income—$0 to $50 K, 40.03%; > $50 K to $100 K, 32.56%; > $100 K to $150 K, 15.15%; and > $150 K, 12.26%.

## Measures

### Outcome Variable—Hypertension

Our outcome of interest was self-reported hypertension (hereinafter hypertension), adapted from the Behavioral Risk Factor Surveillance System questionnaire administered by the Centers for Disease Control and Prevention [28]. Hyper-tension was measured by asking participants whether they were ever told by a doctor that they had high blood pressure. Response options provided were “Yes,” “No,” or “Unsure.”

### Risk Factor Variables

Variation in the experience of racism by geography was measured based on the region where participants lived. This strategy allowed us to capture long-standing and current regional differences in the racial and political climates that affect Black Americans. Participants were from the Northeast, Midwest, South, or West regions of the USA.

The Experiences of Discrimination Questionnaire [29–31], uses global questions rather than behaviorally specific items. The measure focuses on interpersonal and institutional racism in five areas: personal (insults, slurs), education, employment, housing, and police interactions. For this study, a sixth area, law, was added as a modification of the original scale. The items generated two subscales: six items that combined to yield an index of the frequency of experiences of discrimination and twelve items that combined to yield a score for the impact of discrimination. We only report on the frequency of experiences of discrimination scale. Survey items include: “I have experienced racial slurs and insults in social situations,” “I have experienced discrimination at school or in an educational setting,” “I have experienced housing discrimination.” Response options were “never,” “once or twice,” “occasionally,” or “frequently.” For analysis, responses were dichotomized as “never” versus “once or more.” All survey items can be found in Table 1. The alpha coefficient for the total discrimination score was 0.80., for the experience of discrimination subscale it was 0.67, and 0.83 for the impact of discrimination subscale.

We considered personal insults and slurs a form of daily interpersonal racism. Perceived experiences of discrimination in various sectors, including employment, housing, and education, served as a proxy for perceived exposure to institutional-level racism. This approach is consistent with previous research, as shown in a recent review assessing measures of institutional racism [32].

### Covariates

We assessed usual source of care by asking, “Is there a place that you usually go to when you are sick or need advice about your health?” Response options were “There is no place” or “Yes.” We included sex and categorical age in the models as covariates. Sex (female/male) was recorded as a binary variable based on self-reported demographic data and used in statistical analyses. In the discussion, we refer to participants as “men” and “women” to reflect the sociocultural contexts in which racial discrimination is shaped based on gendered social identities and norms [33].

### Analytic Strategy

We examined descriptive statistics for all variables and by hypertension. We assessed significance using chi-square tests for all variables except continuous age, for which we used the independent samples *t* test of significance. Next, we obtained the distribution of indicators of racism by sex, age, and region and evaluated for significance using chi-square. Finally, we conducted logistic regression modeling to determine how indicators of racism across multiple domains were associated with odds of hypertension status and how this association may differ by sex.

We analyzed multiple models; Model 1 was the baseline model, which included only the relationship of all indicators of racism. Model 2 was the adjusted model, which included the covariates usual source of care, age, sex, and education in addition to the racism indicators. We also examined adjusted sex-specific models to determine whether the association of racism with hypertension was different for women (Model 3) versus men (Model 4). Those unsure of a hypertension (*n* = 27) were dropped from the analysis. Finally, we conducted a complete case analysis dropping those from the model missing any data on our variables of interests (*n* = 419). We report significant associations at the 95% confidence level. All analyses were conducted using STATA and SAS.

## Results

### Descriptive Statistics

Demographic characteristics of the sample are presented in Table 2. Women accounted for 51.2%; the mean age was 42.6 years. 67.9% of participants had a high school diploma, or trade, technical, or associate’s degree; and 32.1% had a 4-year bachelor’s degree or higher. Slightly less than half were employed full time (45.5%). This non-representative national sample was older and better educated (bachelor’s and advanced degree holders) but also had a higher percentage who did not have a high school diploma. The percentage without a high school diploma may have been influenced by the age of the sample.

Those with hypertension were more likely to be women, earn between $35,000 and $75,000, have completed education or trade school Beyond high school, and be unemployed. They were also more likely to report having a usual source of care and living in the Western region. The mean age for those who reported hypertension was 50.8 years old.

### Bivariate Analysis

The distribution of experiences of racism indicators by sex, age, and region are presented in Table 3. Compared with women, men were significantly more likely to report experiencing racial insults or slurs, discrimination at school, discrimination in legal situations, and discrimination by police. For example, 67.2% of men reported at least one encounter with discrimination by police, compared with 32.8% of women. Participants between the ages of 30 and 44 years old were significantly more likely to report discrimination in their workplace and by police, compared with other age groups. For example, 33.7% of those aged 30 to 44 years reported at least one encounter with discrimination at their jobs, compared with 23.5% of adults between 18 and 29, 20.2% of those aged 45 to 59 years, and 22.7% of those 60 years and older. People living in the South were significantly more likely to report experiencing racial slurs and insults and discrimination by police compared with those in other regions. Education was also examined in a post hoc analysis. Having associates or trade school level education was associated with higher exposures to discrimination in housing, legal, social, and school settings while the highest education level was associated with job-related discrimination and discrimination in social settings (data not shown).

### Racism and Hypertension

#### Model 1: Unadjusted Model

In the unadjusted model examining the odds of hypertension for those who experienced racism, participants who reported discrimination on the job had higher odds of hypertension. Specifically, those who experienced discrimination once or more on the job had 1.84 times higher odds of reporting a hypertension compared with those who reported never experiencing job discrimination (Model 1; 95% CI: 1.37–2.45). Additionally, those who reported living in the Midwest, South, and West had significantly higher odds of hypertension than those in the Northeast, the region with the smallest number of Jim Crow states in the data. Table 4 shows logistic regression results.

#### Model 2: Includes Covariates

Adding a usual source of care, categorical age, education and sex to the model slightly attenuated the association of odds of hypertension among those who reported discrimination on the job (Model 2; OR_Job_ = 1.44, 95% CI: 1.04–1.99). Moreover, having a usual source of care, and increasingly older age were associated with higher odds of a hypertension (OR_care_ = 1.73, 95% CI: 1.26–2.40; OR_age60_+ = 10.75, 95% CI: 6.92–16.72).

#### Models 3–4: Sex-Specific Models

Similar to Model 2 overall, in sex-specific models, both males and females with a usual source of care and of older age had higher odds of hypertension. Interestingly, females with a college or graduate degree (versus not having completed high school, or having a high school diploma or GED) had lower odds of hypertension (Model 3; OR_Edu_ = 0.52, 95% CI: 0.32–0.89).

## Discussion

In this unique study, we assessed the relationships between hypertension and structural, institutional, and interpersonal racism. We included multiple indicators of racism across multiple domains (e.g., employment, housing, law, police). This process allowed us to examine how various experiences of racism shape hypertension status and, in turn, other cardiovascular diseases.

### Geographic Variation

We found that older age and having a usual source of care were significantly associated with higher odds of hypertension in our overall sample and for women. When we controlled for age in our post hoc analysis, we saw no association between region and self-reported hypertension diagnosis, failing to support our assumption that hypertension would be associated with the region (Southern) most known for Jim Crow Laws, the basis for using region as a proxy for structural racism. Notably, our study examined the region where participants *currently* live versus where they were born or grew up, which may affect how one copes with experiences of racism. Our results suggest that geography may have implications for exposure to discrimination that may vary by sex, highlighting the need to examine the effect of multiple types of racism on health in future studies.

### Types of Racism

In our data, neither being called a racial slur nor experiencing institutional racism in housing, legal situations, or by the police was significantly associated with hypertension in the overall model. This is inconsistent with a 2014 meta-analysis that showed a small, but positive association between perceived racial discrimination and hypertensive status [26]. However, the meta-analysis defined hypertensive status as hypertension diagnosis or use of hypertension medication and confirmed diagnosis by medical chart review. The meta-analysis also stated that perceived discrimination was more strongly associated with nighttime ambulatory blood pressure. Our result is consistent with the results from a 2011 review that showed mixed findings among studies examining interpersonal racism and hypertension. The 2011 review provided evidence that a direct link between individual/interpersonal racism and hypertension was “weak” [34]. Of note, the institutions where people experienced racism changed as they aged. For example, individuals aged 18 to 29 years reported experiencing more frequent institutional racism in school, and those 30 to 44 years reported experiencing more frequent racist experiences on the job, in housing, and in legal situations. This might be expected based on life course demands and suggests the need to consider which racism experiences are more salient for whom.

Interestingly, our data showed that women with a college or graduate degree had lower odds of hypertension than women with a high school diploma, GED, or less than a high school education. This was not true of men, suggesting a need to further explore Black women’s experiences of interpersonal racism by educational attainment. Finally, usual care was associated with higher odds of hypertension for men and women. This may be because those having a usual source of care are more likely to frequent doctors and, thus, are more likely to quickly receive a diagnosis of health conditions.

## Limitations and Future Research

This study has limitations. First, our sample is not nationally representative, and generalizability is limited. Second, our data are cross-sectional, and we cannot infer causality. However, since it is unlikely that hypertension causes people to live in a region or caused women to experience more racial slurs, it is unlikely that our results are subject to reverse causation. Next, a limitation of this study is the use of region-level geographic data, which may obscure important within-region variation; future research using more granular geographic units such as counties or sub-regions is warranted to better capture geographic disparities. We also do not know how long our participants resided in their current region. Duration of living in a region could impact the hypertension risk in multiple ways, for instance access to care and quality of care that may interact with racism experiences.

In addition, individuals who are of mixed racial and ethnic identity might have experiences and attitudes that lead to differences in response. However, because this study used participant self-identification, mixed racial and ethnic identity was not queried. Therefore, we were unable to conduct sensitivity analyses to determine whether or not mixed racial or ethnic identity made any difference to the outcomes observed. This is a potentially important area of exploration for future studies. Similarly, we acknowledge that the use of sex as a binary variable may not fully capture the complexity of gendered experiences. Future research should aim to distinguish more clearly between biological and social influences of health among Black individuals. Another limitation is the use of a self-reported hypertension measure without clinical validation. This may have led to an underestimation, as a recent systematic review and meta-analysis found similarly worded questions are highly specific but have a low sensitivity to detect participants with clinical hypertension [35, 36]. Finally, modifications made to the Experience of Discrimination measure may have affected its reliability which was not assessed prior to use.

## Conclusion and Implications

This study examined the association between multiple domains of racism and the odds of hypertension among a sex- and socioeconomically diverse group of Black people living in the USA. Despite limitations, this study aids in our understanding of how Black people experiencing systems of oppression report racism and how the experience of racism, in turn, influences their health. Our findings underscore the importance of understanding within-group variation and assessing interpersonal, institutional, and structural racism in the same study. Furthermore, the finding that highly educated women report lower odds of hypertension warrants further investigation to understand the mechanisms by which high educational attainment protects against hypertension for women more than men in samples like these.

## Figures and Tables

**Table 1:** Experiences of Discrimination Questionnaire (EDQ) items

Item	Response options
I have experienced discrimination at or on my job	Never, Once or twice, Occasionally, Frequently
I have experienced housing discrimination	Never, Once or twice, Occasionally, Frequently
I have experienced discrimination at school or in an educational setting	Never, Once or twice, Occasionally, Frequently
I have experienced racial slurs and insults in social situations	Never, Once or twice, Occasionally, Frequently
I have experienced discrimination in legal situations	Never, Once or twice, Occasionally, Frequently
I have experienced discrimination by police	Never, Once or twice, Occasionally, Frequently

**Table 2:** Sample characteristics of participants (*N* = 1177)

Characteristic	*N*	% of sample	% reporting HBP	% reporting no HBP	*p* value
Sociodemographic indicators					
Age	1177	42.6 (M)	50.8 (*M*)	37.1(*M*)	<.001
Sex					<.001
Females	603	51.2	45.6	54.4	
Males	574	48.8	34.5	65.1	
Social status indicators					
Income					0.01
Less than $35,000	572	48.6	41.8	58.2	
$35,000—less than $75,000	410	34.8	42.4	57.6	
$75,000 and above	195	16.6	30.8	69.2	
Education					<.001
High School, HS diploma, or GED	307	26.1	33.9	66.1	
Associate’s degree, trade or technical	492	41.8	45.3	54.7	
College or graduate degree	378	32.1	38.6	61.4	
Employment					<.001
Unemployed	444	37.7	50.7	49.3	
Part-time	198	16.8	47.0	53.0	
Full-time	535	45.5	29.0	71.0	
Has a Usual Source of Care					<.001
No	273	23.2	26.4	73.6	
Yes	904	76.8	44.4	55.6	
Interpersonal racism indicators					
Experienced racial slurs or insults					0.06
Never	406	34.5	36.5	63.6	
Once or more	771	65.5	42.2	57.9	
Institutional racism indicators					
Experienced discrimination at school					0.76
Never	529	44.9	39.7	60.3	
Once or more	648	55.1	40.6	59.4	
Experienced discrimination at job					<.001
Never	363	30.8	31.4	68.6	
Once or more	814	69.2	44.1	55.9	
Experienced discrimination in housing					0.10
Never	752	63.9	38.4	61.6	
Once or more	425	36.1	43.3	56.7	
Experienced discrimination in legal					0.61
Never	722	61.3	39.6	60.4	
Once or more	455	38.7	41.1	58.9	
Experienced discrimination by police					0.25
Never	568	48.3	41.9	58.1	
Once or more	609	51.7	38.6	61.4	
Structural racism indicator					
Region					<.001
Northeast	249	21.2	30.5	69.5	
Midwest	218	18.5	42.2	57.8	
South	600	51.0	41.3	58.7	
West	110	9.4	51.8	48.2	

*GED* general education diploma, *HBP* high blood pressure, *HS* high school

**Table 3:** Bivariate analysis of experiences of racial discrimination by sex, age, and region (*N* = 1177)

	% Female	% Male	*p* value	Age, y 18–29	Age, y 30–44	Age, y 45–59	Age, y 60 +	*p* value	Northeast	Midwest	South	West	*p* value
Racial slurs or insults			<.001					0.10					0.01
Never	60.6	39.4		25.9	34.2	20.4	19.5		19.5	18.0	56.2	6.40	
Once or more	46.3	53.7		25.9	33.7	20.5	19.8		22.1	18.8	48.3	10.9	
Discrimination at school			0.03					0.59					0.10
Never	54.8	45.2		24.8	32.9	21.7	20.6		20.2	18.3	54.1	7.4	
Once or more	48.3	51.7		26.9	34.7	19.4	19.0		21.9	18.7	48.5	11.0	
Discrimination at job			0.03					<.001					0.51
Never	55.9	44.1		31.4	34.4	21.2	13.0		22.0	18.7	51.8	7.4	
Once or more	49.1	50.9		23.5	33.7	20.2	22.7		20.8	18.4	50.6	10.2	
Discrimination in housing			0.01					0.19					0.49
Never	54.0	46.0		27.9	33.5	19.6	19.0		20.4	17.8	52.7	9.2	
Once or more	46.4	53.7		22.4	34.6	22.1	20.9		22.6	19.8	48.0	9.7	
Discrimination in law			<.001					0.19					0.58
Never	60.0	40.0		26.0	32.7	19.7	21.6		19.9	19.1	51.8	9.1	
Once or more	37.7	62.6		25.7	35.8	21.8	16.7		23.1	17.6	49.7	9.7	
Discrimination by police			<.001					<.001					0.01
Never	71.0	29.1		25.2	28.5	20.4	25.9		20.3	16.4	55.6	7.8	
Once or more	32.8	67.2		26.6	38.9	20.5	14.0		22.0	20.5	46.6	10.8	

**Table 4:** Odds of self-reported hypertension diagnosis by types of racism in the African American identity, socialization, discrimination, and outcomes project (January–February 2020)

Variable	Model 1: Overall (*n* = 1177)		Model 2: Overall (*n* = 1177)		Model 3: Females only (*n*=603)		Model 4: Males only (*n* = 574)	
	OR	95% CI	OR	95% CI	OR	95% CI	OR	95% CI
Interpersonal racism indicators								
Experienced racial slurs and insults								
Never (ref)	–	–	–	–	–	–	–	–
More than once	1.23	(0.92, 1.65)	1.17	(0.86, 1.60)	1.49	(0.98, 2.28)	0.81	(0.50, 1.31)
Institutional racism indicators								
Experienced discrimination at school								
Never (ref)	–	–	–	–	–	–	–	–
More than once	0.84	(0.64, 1.11)	0.95	(0.71, 1.29)	0.98	(0.64, 1.50)	1.01	(0.65, 1.57)
Experienced discrimination at the job								
Never (ref)	–	–	–	–	–	–	–	–
More than once	**1.84**	**(1.37, 2.45)**	**1.44**	**(1.04, 1.99)**	1.40	(0.90, 2.19)	1.46	(0.90, 2.37)
Experienced discrimination in housing								
Never (ref)	–	–	–	–	–	–	–	–
More than once	1.18	(0.89, 1.57)	1.02	(0.75, 1.39)	1.04	(0.66, 1.62)	1.09	(0.71, 1.69)
Experienced discrimination in law								
Never (ref)	–	–	–	–	–	–	–	–
More than once	1.10	(0.81, 1.49)	1.08	(0.78, 1.50)	0.99	(0.61, 1.616)	1.16	(0.74, 1.84)
Experienced discrimination by police								
Never (ref)	–	–	–	–	–	–	–	–
More than once	**0.64**	**(0.47, 0.86)**	0.85	(0.60, 1.21)	0.89	(0.54, 1.46)	0.83	(0.50, 1.37)
Structural racism indicator								
Region where you live								
Northeast (ref)	–	–	–	–	–	–	–	–
Midwest	**1.71**	**(1.16, 2.51)**	1.39	(0.91, 2.12)	1.65	(0.86, 3.16)	1.24	(0.70, 2.17)
South	**1.59**	**(1.16, 2.19)**	1.38	(0.98, 1.96)	1.67	(0.97, 2.87)	1.15	(0.72, 1.83)
West	**2.46**	**(1.54, 3.92)**	2.02	(0.97, 2.87)	2.02	(0.97, 4.24)	1.76	(0.85, 3.64)
Mediator								
Usual source of care	–	–	**1.73**	**(1.26, 2.40)**	**1.69**	**(1.04, 2.73)**	**1.76**	**(1.12, 2.78)**
Covariates								
Age, y								
18–29 (ref)	–	–	–	–	–	–	–	–
30–44	–		**2.01**	**(1.39, 2.91)**	**2.37**	**(1.30, 4.33)**	**1.77**	**(1.11, 2.84)**
45–59	–	–	**4.08**	**(2.74, 6.08)**	**4.31**	**(2.33, 7.93)**	**3.93**	**(2.29, 6.76)**
60 +			**10.75**	**(6.92, 16.72)**	**12.59**	**(6.86, 23.13)**	**8.84**	**(4.06, 19.27)**
Education								
< High School, HS diploma, GED (ref)			–	–	–	–	–	–
Associate’s degree, trade, or technical school			1.11	(0.80, 1.55)	0.98	(0.60, 1.58)	1.16	(0.72, 1.87)
College or graduate degree			0.85	(0.60, 1.21)	**0.52**	**(0.32, 0.89)**	1.37	(0.83, 2.25)
Males	–	–	1.10	(0.82, 1.48)	–	–	–	–

Model 1. Indicators of racism (insults, discrimination on the job or in school, housing, law, or by police)

Model 2. Indicators of racism plus covariates (usual source of care, age, education, and sex)

Model 3. Females: Indicators of racism and covariates

Model 4. Males: Indicators of racism and covariates

## Data Availability

Access to the data may be granted upon reasonable request to the Principal Investigator, Dr. Vetta Thompson (vthompson22@wustl.edu).
